# A Microglial Function for the Nerve Growth Factor: Predictions of the Unpredictable

**DOI:** 10.3390/cells11111835

**Published:** 2022-06-03

**Authors:** Alexia Tiberi, Simona Capsoni, Antonino Cattaneo

**Affiliations:** 1 Bio@SNS Laboratory of Biology, Scuola Normale Superiore, 56127 Pisa, Italy; alexia.tiberi@sns.it (A.T.); simona.capsoni@sns.it (S.C.); 2 Institute of Neuroscience, National Research Council (CNR), 56124 Pisa, Italy; 3 Section of Human Physiology, Department of Neuroscience and Rehabilitation Biomedical and Specialty Surgical Sciences, University of Ferrara, 44121 Ferrara, Italy; 4 Rita Levi-Montalcini European Brain Research Institute (EBRI), 00161 Roma, Italy

**Keywords:** microglia, nerve growth factor, neurotrophin, neuroimmune communication

## Abstract

Microglia are the only immune cell population present in the brain parenchyma. Their vantage position in the central nervous system (CNS) enables these myeloid cells to perform the most disparate of tasks: from the classical immune functions of fighting infections and surveilling the extracellular space for pathogens and damage, to sculpting the neuronal circuitry by pruning unnecessary synapses and assisting neurons in spine formation, aiding in the maintenance of brain homeostasis. The neurotrophin field has always been dominated by the neurocentric view that the primary target of these molecules must be neurons: this holds true even for the Nerve Growth Factor (NGF), which owes its popularity in the neuroscience community to its trophic and tropic activity towards sensory and sympathetic neurons in the peripheral nervous system, and cholinergic neurons in the CNS. The increasing evidence that microglia are an integral part of neuronal computation calls for a closer look as to whether these glial cells are capable of responding directly to NGF. In this review, we will first outline evidence in support of a role for NGF as a molecule mediating neuroimmune communication. Then, we will illustrate some of those non-immune features that have made microglial cells one of the hottest topics of this last decade. In conclusion, we will discuss evidence in support of a microglial function for NGF.

## 1. Rediscovering Old Concepts

It is not unheard of in the field of neuroscience for a concept to go “forgotten” only to be brushed anew when new evidence comes forth. Such was the case for microglia’s special affinity toward spines: the first manuscript to ever intimate how microglia could somehow participate in the workings of neuroscience’s favorite specialization—the synapse—was published during the late sixties. This seminal work identified a peculiar activity carried out by activated microglia after facial nerve injury, the displacement of synaptic terminals from regenerating motor neurons [1]. This particular kinship got somewhat lost at the fringes of neuroscience until Dr. Beth Steven, working as a postdoc at Stanford School of Medicine, gave it new life some 40 years later, when she published the first of what would then become a cascade of papers suggesting microglia as the cellular elements capable of physically “pruning” synapses [2].

The Nerve Growth Factor (NGF) has had a similar history. That neurotrophins could have effects outside their canonical activity on neurons has been known for quite some time. Rita Levi-Montalcini herself, in her Nobel Lecture of 1986, wonders about the intricate and unpredictable nature that has been intrinsic to the study of NGF:

*“Predictions of the unpredictable are encouraged by the same history of NGF, which may be defined as a long sequence of unanticipated events, which each time resulted in a new turn in the NGF uncharted route and opened new vistas on an ever-changing panorama. One can at present only predict where future developments are most likely to occur. The main causes of unpredictability of the findings reside in the intricacy of the new surroundings where NGF is moving—the CNS and the immune system—rather than in NGF itself. The enormous complexity of these two networks, which on the basis of recent findings are closely interrelated and influence each other through bidirectional signals, opens endless possibilities for NGF activation of distinct repertoires of cells belonging to one or the other system.”* [3].

The discoverer of NGF herself had thus already guessed and pursued the idea that NGF could act as a means of communication between two very different systems—the nervous and the immune system, to the point that the definition of NGF itself (Nerve Growth Factor) may be a misnomer. This peculiar activity of NGF is, though, often forgotten when it comes to its role in the central nervous system (CNS), where it is mostly known for its trophic and tropic activity on cholinergic neurons of the basal forebrain (BFCNs). But as the microglia field keeps evolving at impossible rates, unveiling these tiny cells as important players in plasticity mechanisms of all sorts in the CNS, NGF acquires new allure, precisely for its ability to serve two kings. Indeed, if the effect of NGF on peripheral immune cells has been established in the literature, a question ultimately remains: how does this particular activity of NGF reflect on the brain’s very own immune cell, microglia?

## 2. Nerve Growth Factor: Caught in between Neuroscience and Immunology

NGF is the first and one of the best-characterized proteins of the family of neurotrophins. After nearly 70 years from its discovery, interest in this particular molecule has never subsided precisely because of its pleiotropic activity: its influence spans from neuronal development and survival, to neurodegenerative and autoimmune diseases. Like all neurotrophins, NGF can interact with two distinct classes of receptors: a tyrosine kinase receptor (Trk)—TrkA, in the case of NGF—and the so-called low-affinity neurotrophin receptor p75^NTR^, shared among different neurotrophins. NGF is first synthesized as a precursor form—proNGF—which possesses a higher affinity for p75^NTR^ and owns its own peculiar signaling properties. The protein undergoes proteolytic cleavage to yield mature NGF, switching its affinity toward TrkA [4]. The complex signaling mechanisms via these receptors go beyond the scope of this review and have been extensively described in the existing literature [5,6,7].

The neurotrophin system is ancient, as orthologues of neurotrophins, p75^NTR^, and Trk receptors are found in invertebrates as diverse as sea urchins, mollusks, and roundworms [8]. Therefore, this family has had half a billion years of evolution to develop extraordinary complexity in function. Neurotrophins, as a family, are best known for their effect on neurons: the term itself, *neurotrophin*, leaves little to the imagination. Its etymology comes from the word νεῦρον (nevron), which means *nerve*, and τροϕή (trofon), which means *nutrition*, implying in a very literal manner their core activity in supporting neuronal survival. Indeed NGF is no different: it was first described for its ability to promote growth and differentiation of sensory and sympathetic neurons in the peripheral nervous system (PNS) [9]. NGF does not simply act as a phasic—ON/OFF—type of molecule, as it is involved in the tonic control of neurotransmitter and neuropeptide synthesis: for instance, in sympathetic neurons, the production of norepinephrine is regulated by NGF through selective induction of tyrosine hydroxylase (TH) [10]. In dorsal root ganglia, the expression of neuropeptides by primary sensory neurons is under the control of the neurotrophin [11] such that in vivo deprivation of NGF, as a result of nerve transection or anti-NGF treatment, causes a marked decrease in Substance P (SP) and Calcitonin Gene-Related Peptide (CGRP) synthesis [12].

In the periphery, NGF signaling is also involved in nociception: injecting NGF into healthy human skin can produce localized pain and hyperalgesia that develops within minutes [12,13]. Intravenous injections of low doses of NGF (1 μg/kg) result in myalgia—a widespread, deep musculoskeletal pain affecting proximal body regions and reminiscent of the sensory disturbances observed frequently with mild infections. Accordingly, mutations in the NGF gene cause a disease characterized by pain insensitivity called Hereditary Sensory and Autonomic Neuropathy type V (HSAN V) [14].

In the CNS, basal forebrain cholinergic neurons (BFCNs) are highly dependent on NGF for their survival, and they can obtain the neurotrophin through retrograde transport from target areas [15,16]. Indeed, by cutting the fimbria—the physical connection between BFCNs and their target areas—a degeneration of cholinergic neurons occurs, one that can only be rescued via NGF administration [17]. Since NGF cannot cross the blood-brain barrier (BBB) [18], the NGF needed for cholinergic neurons’ survival is produced *in loco* by the neocortex and the hippocampus, known targets of cholinergic neurons [19,20], primarily by GABAergic interneurons [21]. Furthermore, tampering with NGF expression either genetically or via the expression of neutralizing antibodies for either TrkA or mature NGF results in atrophy of cholinergic neurons accompanied by memory and learning deficits [22,23,24,25]. NGF also behaves as a neuromodulator of cholinergic activity: specifically, NGF is known to enhance acetylcholine (ACh) release from BF [26], medial septum, and diagonal band of Broca cholinergic neurons [27] in a p75^NTR^ dependent manner.

It is worth noting, though, that the expression of anti-NGF antibodies in the adult brain of transgenic mice, determines a progressive neurodegeneration with synaptic and behavioral deficits, and with neurodegeneration hallmarks reminiscent of an Alzheimer’s disease-like phenotype, an effect much broader than what one would expect based on the action of these antibodies exclusively on the cholinergic system [23].

Indeed, the broad activity of NGF does not end within the confines of the nervous system. As often happens in biology, nature never misses a chance to reuse molecules for disparate functions. In fact, NGF does not simply act on neurons, but it can also affect another entirely separate population: immune cells. The need for such a Janus-faced activity of NGF on nervous and immune cells should not come as a surprise. The nervous and immune systems indeed share similar functions: they are both in charge of maintaining homeostasis and they both react to and translate external stimuli [28]. To achieve such harmony in integrated responses, neuroimmune communication requires close anatomical connections and functional interactions based on shared receptors and common pathways [29,30]. If the effect of NGF on neurons is established, the other side of the neuroimmune coin resides in considering the evidence for an immune function of NGF.

A clue in favor of an immune role for NGF comes from the fact that its levels greatly increase after acute or chronic inflammatory insults such as multiple sclerosis, chronic arthritis, systemic lupus erythematosus, and mastocytosis [31,32], a feature NGF shares with cytokines and strongly suggests an immunomodulatory activity. A particularly compelling case is also made by the presence of NGF receptors—or the expression of NGF itself—in a wide variety of immune cells. The existing literature on NGF and immune cells (see Table 1) paints the picture of autocrine and paracrine loops modulating immune responses that see NGF as a key player: for instance, mast cells have been shown to both secrete and respond to NGF [33,34]. Moreover, NGF acts as a proliferation and differentiation factor for B- and T-lymphocytes, modulating immunoglobulin (Ig) production [35,36,37]. Of interest for the purpose of this review is the body of research papers on the peripheral immune cells that closest resemble microglia: macrophages (Mφs). One report shows that Mφs respond to NGF by secreting TNF-α [38]. Another describes NGF as an autocrine factor essential for the survival of Mφs infected with HIV [39]. Interestingly, in a more recent paper, NGF stimulation of Mφs increased membrane ruffling, calcium spiking, phagocytosis, and growth factor secretion. In contrast, proNGF induced podosome formation, increased migration, suppressed calcium spikes, and increased neurotoxin secretion [40]. This last piece of evidence shows that the effect of NGF on myeloid cells is pleiotropic, spanning from motility to function: we will later see how important motility is for the myeloid cells of the brain, microglia, so keep this information in mind.

Some pieces of evidence bringing the world of NGF and immunity together reside also in the evolutionary ontology of NGF receptors. For instance, the NGF receptor p75^NTR^ is a member of the tumor necrosis factor receptor superfamily: this family includes many immunologically relevant receptors, such as TNFα, FAS, CD40, which share structural and sequence homology, and thus point towards a common ancestral origin for these proteins and the NGF receptor [41]. Better yet, an interesting body of literature has found an unforeseen receptor for neurotrophins in *Drosophila Melanogaster*: Toll receptors [42,43]. These receptors and their mammalian counterparts, Toll-like receptors, are primarily known for their role in immunomodulation. Thus, these evolutionary links between immunity and neurotrophins reinforce the idea that these two seemingly unrelated systems are much more intimately connected than one could have imagined.

Prescient as ever, Rita Levi-Montalcini herself, in a review from 1996, redefines NGF as part of a larger family of proteins together with cytokines, coining the word “neurokine” [44].

Accordingly, considering NGF involvement in both neuronal and immune processes, it is intuitive to think of NGF as a molecule mediating the orchestra of immune and nervous systems [45]. With that said, it is thus conceivable that also the immune cells of the CNS, microglia, could be a target of NGF. Before laying out the data, let us consider some of those features that have made microglia one of the hottest topics of the last decade in neurobiology, and that make it particularly interesting to investigate how to modulate the function of these cells, both in health and disease.

**Table 1:** NGF-responsive immune cells.

Cell type	Main Finding	Ref.
**Monocyte/Macrophages**	LPS increases both NGF and NGF receptors expression	[46]
	NGF decreases the inflammatory response	[47]
	NGF dependent increase in CXCR4 expression and chemotactic response	[48,49]
	NGF dependent increase phagocytosis, enhanced parasite-killing activity and IL-1	[50]
	NGF dependent increase TNF-α, IL-8 secretion	[38]
	NGF is an autocrine factor involved in survival	[39]
	NGF and proNGF differentially regulate macrophage phenotype	[40]
**T cells**	Expression of NGF mRNA	[51]
	Activated T cells express NGF and TrkA	[52]
	NGF expression increased in T cells after injury | optic nerve crush	[53]
	Expression of NGF receptors	[54]
**B cells**	NGF and TrkA expression	[55]
	NGF dependent differentiation and increase IgM production	[37]
	Increase in proliferation	[56]
	Increased NGF expression after stimulation	[57]
	NGF expression and secretion	[34]
**Mast cells**	NGF dependent increase in tryptase, IgE receptors and histamine	[58]
	NGF dependent increase in cyclooxygenase2 (COX2) and prostaglandin D2	[59]
	Mast cells proliferate in the presence of NGF	[33]
	Mast cells express NGF	[34]
	NGF dependent IL-6 induction, decrease TNF α	[60]
	NGF induces degranulation	[61]
	NGF dependent histamine release	[62]
	NGF dependent increase in chemotaxis	[63]

## 3. Microglia: A Duplicitous Nature

There are two key functional features that define microglia and they mirror their duplicitous nature as immune cells and as cells of the CNS: immune defense and maintenance of CNS homeostasis. As for their immunological functions, microglia constantly sample their environment, scanning and surveying for signals of external danger [64,65], such as those from invading pathogens, or of internal danger generated locally by damaged or dying cells [66]. Detection of such signals initiates a program of microglial responses that aim to resolve the injury, protect the CNS from the effects of the inflammation, and support tissue repair and remodeling [67].

On the other hand, microglia are profoundly molded by the surrounding CNS environment [68]: in mice, after the engraftment in the brain parenchyma, microglial cells progressively acquire a more ramified morphology and reach the adult pattern of homogeneous tiling during the second postnatal week [69]. During this maturation process, microglia undergo different phases of differentiation that rely on signals derived from the maturing CNS, the gut microbiome, sexual identity, and inflammatory molecules [70,71,72]. Transcriptomic studies have highlighted different signatures for microglia from female and male mice, a sexual dimorphism that appears to be a long-lasting reprogramming, conserved after grafting [73].

As per their origin, it has now been conclusively proved that microglia arise from the yolk sac (YS)—an extraembryonic mesoderm site of hematopoiesis—and enter the brain as amoeboid primitive macrophages prenatally, persisting and proliferating in the CNS into adulthood [74,75]. In the YS, the earliest primitive wave of hematopoiesis occurs at embryonic day (E) 7.5 in mice, and this generates nucleated red blood cells and macrophages that go on to colonize the whole organism. These myeloid cells start invading the neuroectoderm at E9 before the closure of the BBB that will restrict any further access to the brain parenchyma. At these early stages of development, in the brain, neural progenitors are giving rise to the first neuronal cells, and only later on they will generate oligodendrocyte and astrocytes, making microglia the main glial population during a good part of the life of the embryo. Outside of the CNS, YS-derived macrophages are gradually replaced by circulating monocytes coming from the later fetal and definitive bone-marrow hematopoiesis [76,77]. Conversely, microglia, which are closed off from the circulation by the BBB, will act as a standalone population, and will only grow in numbers by self-renewal, at least under steady-state conditions. As immune cells, microglia not only retain the ability to recognize programmed cell death and engulf dying or dead cells [78,79] but there is multiple evidence that they can themselves actively initiate a cell death program. In cerebellar slices, microglia induce apoptosis in Purkinje neurons by releasing superoxide ions [80]. Another signaling cascade by which microglial cells interact with neurons to induce cell death is mediated by tumor necrosis factor alpha (TNF-α) [81]. On the other hand, microglial cells can also positively influence development by promoting neural precursor cell proliferation and survival [82,83].

Microglial cells are the professional phagocytes of the brain and as such, they are capable of eliminating entire cells or cellular substructures [84]. But if one had to pinpoint the origin of the renewed interest in microglia, it would be for their involvement in synaptic pruning. The term “synaptic pruning” indicates the elimination of weak synaptic connections, a process that contributes to homeostatic plasticity. The first evidence that microglia might be involved in such activity comes from two concomitant studies. One, carried out in the lab of Dr. Beth Stevens, identified microglia as essential participants in the elimination of the presynaptic inputs from the retinal ganglion cells into the dorsal lateral geniculate nucleus [2,85]. The other, from Cornelius Gross’ lab in Monterotondo, found that PSD95 positive puncta (so a proxy for postsynaptic terminals) could be found inside microglial cells and that such an engulfment required the microglial fractalkine receptor CX3CR1 in the mouse hippocampus at P15 [86]. The “eat me” signals proposed by the authors of the Stevens paper were the complement proteins C1q and C3, which supposedly tag the synapses to be engulfed. Conversely, CD47 has been later identified as one of the “don’t eat me” signals, protecting synapses from excess pruning [87]. These positive and negative signals to microglia don’t come out of the blue: they are shared with macrophages in the periphery, where complement has opsonizing properties to aid phagocytosis, and CD47 is known to be expressed by cancer cells precisely to evade immune detection [88].

Not all tools in the microglial belt seem to involve the need for a physical phagocytosis: a report identified the microglial TWEAK and neuronal Fn14 to be the duo driving the refinement of retinogeniculate connectivity through a non-phagocytic mechanism in a phase of neural development driven by sensory experience [89].

If microglia are then involved in spine elimination, it was also shown that these cells also contribute to spine formation. For instance, in the somatosensory cortex of a P8 mouse, microglia contact with dendrites can induce filopodia formation [90]. This facilitatory role of microglia in synaptic circuit remodeling and maturation was further confirmed in organotypic hippocampal cultures, where dynamic microglia-synapse interactions induced presynaptic partial phagocytosis (trogocytosis) and formation of postsynaptic spine head filopodia by microglial contact [91].

If the involvement of microglia in synaptic pruning during development is now an established fact, there is also increasing evidence that these cells have a deep connection to synapses in the adult healthy brain in the framework of neuronal plasticity. Microglia are highly motile cells and continuously scan the environment with their ever-moving processes [64]. By using in vivo two-photon imaging, it was shown that microglial processes make direct contact with synapses at a frequency of about once per hour. Contacts were activity-dependent and decreased in frequency upon reductions of neuronal activity [92]. In the visual cortex, it was demonstrated that microglia interact with axonal terminals and dendritic spines, and this interaction depends on changes in neuronal activity [93]. Light deprivation makes microglia less motile and changes their localization preference to the vicinity of a subset of larger dendritic spines that persistently shrink, while light re-exposure reverses these behaviors. Motility and morphology of these cells are thus a direct correlate of their action on neurons, as it is apparent that, at least for some features of microglia-neuron communication, contact is required.

This access to spines and sensitivity to neuronal activity must have an impact on synaptic function and plasticity, which requires neurons and microglia to speak a common language in order to achieve homeostasis. Many indeed are the signaling mechanisms that are now known to contribute to neuroimmune communication (see [94] for a comprehensive review). The most obvious candidates for such a role are, of course, immune molecules. Indeed, many cytokines are already known to be involved in plasticity mechanisms: if we have previously mentioned the complement family and CD47 as known modulators of the process of pruning, emerging evidence demonstrates how signaling in immune pathways, related to microglia, can affect synaptic strength. For instance, the application of the chemokine CX3CL1, whose receptor CX3CR1 is exclusively expressed in microglia, on brain slices causes depression of synaptic transmission [95]. Moreover, it was shown that microglia contact on spines increases synaptic activity, while microglial depletion decreases the synchronization of neuronal populations [96].

Interestingly, not all neuroimmune pathways involve canonical immune molecules. Recently, two new studies demonstrated that microglial dynamics are deeply influenced by norepinephrine signaling: noradrenergic tone seems to be suppressing microglia process surveillance during wakeful state [97,98]. Moreover, microglia can also respond to cholinergic tone via the α7 nicotinic acetylcholine receptor [99,100]. Most importantly for this review, recent work [101] has identified a potential role for microglial brain-derived neurotrophic factor (BDNF), one of the members that along with NGF constitutes the neurotrophin family. In that paper, selective deletion of microglia or genetic removal of microglial-derived BDNF in mice at postnatal day 30 caused deficits in multiple learning tasks and a significant reduction in motor-learning-dependent synapse formation [101]. So, if the wall between the neurotrophin field and microglia has already been abolished, let’s delve into the data regarding specifically NGF.

## 4. A Microglial Function for the Nerve Growth Factor

If microglia are to sense NGF and respond to it, it stands to reason that these cells must have NGF receptors. Indeed, the majority of reports point to TrkA as the receptor mediating the effect of NGF on microglia: a first account revealed that NGF was able to increase microglial chemotactic behavior in cell culture, a response that was sensitive to K252a, a tyrosine kinase inhibitor, suggesting the involvement of TrkA [102]. Similarly, it has been reported that NGF can also promote microglial proliferation via TrkA [103]. Conversely, some work shows that NGF can also act on microglia via p75^NTR^, by inhibiting their expression of MHC class II molecules [104].

In a 2018 paper from our lab [105], we also reported that microglia in vitro and ex vivo express TrkA, as demonstrated by binding of the TrkA-specific monoclonal antibody MNAC13 [106], and p75^NTR^—and display the proper signaling machinery to respond to the neurotrophin. Interestingly, NGF was able to profoundly modulate microglial gene expression towards an anti-inflammatory neuroprotective phenotype: this was paralleled by functional modifications of their motility and phagocytic behavior, the latter specifically towards soluble molecules, including soluble forms of Aβ. Ex vivo, patch-clamp recordings from microglia showed that NGF triggers an outward current in these cells, suggesting a direct effect. Moreover, we could observe the same increase in microglial phagocytic behavior after NGF administration in acute brain slices, suggesting the translatability of our in vitro data to an in vivo setting. Another report later identified similar TrkA-dependent anti-inflammatory properties of NGF on microglia after LPS administration [107]. Indeed, NGF could reduce the secretion of pro-inflammatory cytokines such as TNFα, IL-6, and IL-1β and attenuate nitric oxide production. Interestingly, the authors also show how this anti-inflammatory effect of NGF is accompanied by profound metabolic changes in microglia, such as reduced glucose uptake and decreased glycolytic activity. Thus, it appears that NGF is capable of attenuating the pro-inflammatory response of microglia via TrkA. In some measure, this has also been observed *in vivo*: treating an Alzheimer’s disease mouse model, the 5xFAD, with a mutein of NGF that behaves as a TrkA-biased agonist greatly ameliorates cognitive symptoms via a mechanism that involves microglial cells and requires the necessary modulation of important immune-related chemokines and cytokines [108]. Consistent with this body of literature suggesting an anti-inflammatory role for NGF in the brain, getting rid of mature NGF via the expression of selective antibodies causes an early and strong neuroinflammatory response in the brain of the AD11 transgenic mouse model of neurodegeneration [109]. What remains to be determined is whether NGF-TrkA signaling in microglial cells is relevant in pathological conditions only, or whether there is also a homeostatic mechanism of neuroimmune communication relying on this pathway in health.

That TrkA activation could bring about anti-inflammatory effects on microglia was also demonstrated for another TrkA ligand: Dehydroepiandrosterone (DHEA). DHEA is an abundant circulating steroid hormone in humans, present also in brain tissue. Interestingly, DHEA was shown to regulate microglial inflammatory responses through phosphorylation of TrkA and subsequent activation of a pathway involving Akt1/Akt2 and cAMP response element-binding protein [110]. The paper also identifies the downstream mediator of TrkA anti-inflammatory activity, the histone 3 lysine 27 (H3K27) demethylase Jumonji d3 (Jmjd3), an epigenetic regulator known to be involved in aging-related diseases [111]. Further studies are needed to evaluate if Jmjd3 is also involved in mediating NGF-driven anti-inflammatory changes in microglia.

The other side of the story resides in the fact that there also seems to be a function for microglia-derived NGF. For instance, the precursor form of NGF, proNGF, expressed by microglial cells was shown to be the cause of cell death in the developing retina via a mechanism involving p75^NTR^ [112,113]. Another piece of evidence indicates that microglial-derived proNGF can negatively influence the differentiation of cholinergic neurons in vitro [114]. The expression of NGF by these brain myeloid cells seems to be highly dependent on their inflammatory profile: NGF expression increases after an inflammatory stimulus such as LPS and it is dependent on the activity of the transcription factor regulator NF-κB [115]. Similarly, increased expression of NGF was detected in microglial cells after axonal injury in the corpus callosum in vivo [116] or after stimulation with agonists for A2a-receptors for adenosine [117], whose extracellular concentration is known to increase after injury [118]. Thus, it seems that microglial cells produce NGF specifically during pathological situations.

Still, there is much to be understood about the role of microglial-derived NGF. For instance, there is the matter of form: namely, does microglial-derived NGF exist as the precursor form only, proNGF, or does it carry out its functions also in the mature form? Or better said, how does microglial-derived NGF contribute to the balance between death and survival that is implemented by p75^NTR^ and TrkA? Many of the references cited above detect specifically proNGF in pro-inflammatory microglia, and accordingly report activation of the apoptotic p75^NTR^ signaling pathway in surrounding cells. ProNGF secreted by microglia could thus constitute a negative byproduct of microglial activation, contributing to neurodegenerative processes. In any case, since neurotrophins can also be cleaved in the extracellular milieu [119], once microglial-derived NGF is in the extracellular space this could be processed into its mature form thus contributing to TrkA signaling as well, aiding neurons in plasticity mechanisms that require TrkA homeostatic activation [120]. Therefore, it would be of the utmost interest to understand how the balance between microglial-derived NGF and proNGF contributes to disease progression. Indeed, the proNGF/NGF balance—though not related to microglia cells in particular—has been heavily implicated in the development of neurodegenerative disorders: in the cortex of Alzheimer’s disease patients, proNGF levels are increased and there is a striking reduction of TrkA expression [121,122]. Consistently, tipping the scale towards proNGF *in vivo*—by expressing a cleavage-resistant proNGF—causes memory impairment and cholinergic deficits in a transgenic mouse line [123]. Thus, understanding how microglial cells contribute to this balance could be extremely relevant for neurodegenerative disorders and could provide a novel therapeutic target. In order to get there, future work on the topic will need to discriminate between the mature and precursor forms of NGF. The importance of this balance brings us back to the anti-inflammatory properties of NGF mentioned above: the ability of microglial cells to produce their own NGF could endow them with an autocrine anti-inflammatory ability, capable of keeping inflammation in check.

**Figure 1:**
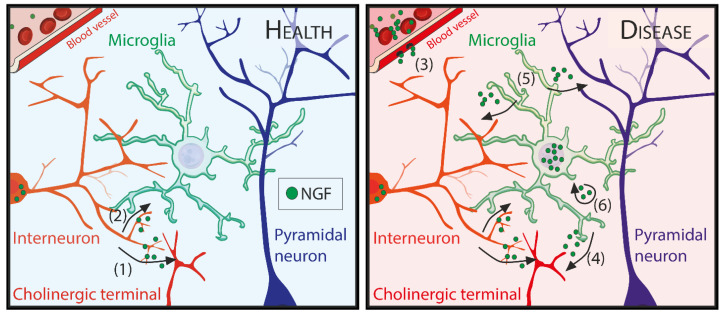
Hypotheses on the activity of NGF on microglia *in vivo*. In health (left panel), (1) NGF is produced by interneurons and should act as a tonic modulator of cholinergic activity. Microglia, which have access to the synaptic cleft, (2) could possibly sense a spillover of neurotrophin and participate in the homeostatic changes brought about by the increase in arousal upon NGF effect on acetylcholine production, affecting neuronal activity. In disease (right panel), concentrations of NGF increase systemically under inflammatory conditions and (3) might reach the brain parenchyma if the BBB is disrupted. Moreover, NGF/proNGF can also be produced by activated microglia to act on (4) TrkA receptors that are exclusively expressed on cholinergic neurons or, (5) as proNGF, on p75^NTR^ expressed by other neuronal populations. Microglia could themselves be affected (6) by the increase in NGF as suggested by Rizzi et al. 2018 [105] with a paracrine or autocrine loop that could possibly help keeping the inflammation in check avoiding the neurotoxic side effects of inflammation.

Given the literature presented in this review, we can hypothesize two situations (Figure 1). In the healthy brain, in which the levels of immune-derived NGF are low or in any case restricted by the BBB, NGF is mainly produced by interneurons to sustain arousal via the modulation of cholinergic activity. At the level of the synapse where the neurotrophin is secreted, microglia could have direct access to NGF and be able to sense and react to it, locally and acutely. Whether and how the ability of microglia to sense NGF is relevant in healthy situations remains to be determined. Still, if it were, microglia could then modulate neuronal activity in the local circuit through many of the mechanisms of neuroimmune communication mentioned in the “*Microglia: a duplicitous nature*” section of this review.

On the other hand, in an inflammatory state such as injury or neurodegeneration, NGF levels rise both systemically and possibly also locally via increased NGF expression by microglia. It is important to note that during disease the BBB is often compromised [124], making it possible for NGF produced by immune cells in the periphery to reach the brain. Moreover, BBB disruption would also allow for infiltration of other NGF-producing cells in the brain parenchyma, such as T-lymphocytes, a common occurrence during disease [125,126,127]. In this situation, microglial-derived NGF could mediate apoptotic death of injured neurons via p75^NTR^ activation in the form of proNGF, or it could act in its mature form both in a paracrine manner on nearby TrkA positive neurons or in an autocrine loop on microglia to reduce the inflammation. Since TrkA is exclusively expressed in cholinergic neurons [128], microglial-derived mature NGF could then possibly affect the modulation of arousal levels impinging on cholinergic activity directly in a brain-wide manner. As proNGF, it could otherwise impinge on p75^NTR^ which is expressed on both neurons and astrocytes [129,130]. In this context, the ability of microglia to detect the cholinergic tone mentioned above could be important, providing a feedback on the arousal state in the brain. This is an hypothesis that will have to be tested by modifying in vivo the ability of microglial cells (1) to respond to NGF or (2) to secrete NGF itself.

In any case, this dual mode of action—one local and acute, the other systemic and broad—is not unusual for immune-related molecules in the CNS. Let’s take for example TNFα. This classic inflammatory cytokine is mostly known for its role in regulating the immune system. Expressed by immune cells at high levels during inflammatory events, it is one of the most powerful pro-inflammatory molecules [131]. TNFα though has been shown to have a most interesting role in the healthy brain. This molecule, which can be secreted also at low concentrations by glial cells in the healthy brain, is a powerful regulator of homeostatic plasticity [132,133]. As already highlighted in the second paragraph of this review, Rita Levi-Montalcini herself defined NGF as a neurokine, for the similarities in behavior and function that the neurotrophin shows towards molecules that are known immune mediators, chemokines and cytokines. Comparing NGF and TNFα, in the healthy and in the diseased brain, might not be a far-fetched deal then, when one and the other are likely to carry their functions in a very similar fashion, depending on the context and level of expression.

## 5. Conclusions

The microglia field is gaining considerable traction because of the now recognized effect of the immune system on the healthy and the pathological brain. In this context, the discovery of new (and old) molecules capable of modulating immunity is of the utmost priority. This is particularly evident in the field of Alzheimer’s disease, in which microglia have risen as determining contributors to the pathogenesis and progression of the disease [111,134]. The properties of NGF highlighted in this review could be exploited to harness the brain’s innate immunity as a safe *in loco* neuroprotective agent. Thus, the neurotrophic and neuroprotective actions of NGF in the CNS, that have classically been considered to be restricted to NGF target neurons, i.e., basal forebrain cholinergic neurons, are potentially much broader, since via the interaction of NGF with microglia, they could be indirectly extended to a much larger class of neurons. This hypothesis, though, remains to be proven. To truly uncover how NGF and microglia interact in an in vivo physiological and pathological setting, there is much need for in vivo work that tackles the issue systematically by means of murine models and imaging in vivo. The glia field is expanding in uncharted routes: broad projection systems, such as the noradrenergic and cholinergic systems, are just now starting to be connected to glia (both microglia and astrocytes). If we had to predict a possible future for microglial cells, it would be precisely in the mediation of such broad changes of neuronal states—arousal, wakefulness etc. Thus, NGF and other neuroimmune molecules capable of mediating neuroimmune communication might just prove to be valuable tools to modulate the response of microglial cells, particularly in situations of disease in which the homeostatic mechanisms brought forth by these cells go awry.
